# Supplementary material for: Of shared homes and pathways: free-ranging dog movement and habitat use in a human-wildlife landscape in India
Source: Mov Ecol. 2026 Feb 17;14:14. doi: 10.1186/s40462-026-00632-2 (PMC12961862; doi:10.1186/s40462-026-00632-2)
Supplement: Supplementary file 2 — Supplementary Material 2 [file 40462_2026_632_MOESM2_ESM.docx]

Table S1. Number of GPS points for each individual dog in each habitat type. Dog names are provided to facilitate reference to the Movebank database.

| ID | Name | Agricultural  land | Deciduous forest | Fallow/  scrubland | Settlement | Thorn forest | Water | Total |
| --- | --- | --- | --- | --- | --- | --- | --- | --- |
| D1 | Appu | 22 |  | 45 | 1102 |  |  | 1169 |
| D2 | Biscuit | 36 |  | 27 | 2365 |  | 18 | 2446 |
| D3 | Brown | 561 |  | 45 | 2176 | 1 |  | 2783 |
| D4 | Burger | 301 | 202 | 10 | 1638 | 21 |  | 2172 |
| D5 | Chan | 490 | 30 |  | 1303 |  | 51 | 1874 |
| D6 | Kamaru | 36 |  | 113 | 731 | 25 | 1 | 906 |
| D7 | Kutti | 9 | 1 | 3 | 736 | 1 | 7 | 757 |
| D8 | Mani | 44 |  | 3 | 2127 |  |  | 2174 |
| D9 | Meenu | 1 |  | 3 | 643 |  |  | 647 |
| D10 | Puppy | 134 | 203 | 26 | 622 | 219 | 3 | 1207 |
| D11 | PuppyMS | 35 |  |  | 1105 |  |  | 1140 |
| D12 | Simba | 8 |  | 64 | 1428 | 1 |  | 1501 |
| D13 | Sophie | 19 |  | 19 | 581 |  |  | 619 |
| D14 | Sundari | 28 |  | 5 | 602 |  |  | 635 |
| D15 | Vellian | 18 |  | 208 | 1986 | 5 | 30 | 2247 |
|  |  | 1742 | 436 | 571 | 19145 | 273 | 110 | 22277 |

Table S2. Table of median home/activity range from studies across different countries (adapted from Ladd et al., 2023; rows marked with an asterisk are new additions to the table.)

| Study | Location | Method | Median home/activity range (ha) | Tracking  duration | Tracking  method |
| --- | --- | --- | --- | --- | --- |
| Meek (1999) | Australia | MCP isopleth, outliers excluded | 72.50 | 15 months | VHF radio tracking |
| Van Kesteren et al. (2013) | Kyrgyzstan | Characteristic hull polygon | 2.26 | Mean 20 h | GPS tracking |
| Sparkes et al. (2014) | Australia | MCP 100% isopleth, forays excluded | 37.47 | 7 days | GPS tracking |
| Dürr and Ward (2014) | Australia | MCP 95% isopleth | 3.60 | Mean 50 h | GPS tracking |
| Ruiz-Izaguirre et al. (2015) | Mexico | Kernel density | 16.10 | 45 days | VHF radio tracking |
| Kennedy et al. (2018) | Australia | MCP 100% isopleth, forays excluded | 8.88 | 3–5 days | GPS tracking |
| Muinde et al. (2021) | Kenya | MCP 95% isopleth | 9.30 | 5 days | GPS tracking |
| Saavedra-Aracena et al. (2021) | Chile | Kernel density | 19.20 | Mean 20.5 days | GPS tracking |
| Warembourg et al. (2021) | Chad  Guatemala  Indonesia  Uganda | Biased random bridge, 95% isopleth | 7.70  5.70  5.60  5.70 | Median 60.3 h | GPS tracking |
| Ladd et al. (2023) | Cambodia | MCP 95% isopleth | 91.40 | Mean 25.5 days | GPS tracking |
| Wilson-Aggarwal et al. (2021)* | Chad | Continuous time movement models, AKDE (95% | 54 (dry season)  31 (wet season) | Mean 37 days | GPS tracking |
| Dürr et al. (2017)* | Australia | Biased random bridge, 95% isopleth | 4.48 | 2-16 days | GPS tracking |
| Hudson et al. (2017)* | Australia | Biased random bridge, 95% isopleth | 5.59 | Median 13 days | GPS tracking |
| Molloy et al. (2017)* | Australia | Biased random bridge, 95% isopleth | 3.1 | Median 25 hours | GPS tracking |
| Schuttler et al. (2022)* | Chile | Continuous time movement models, AKDE (95% | 15.8 (spring)  21 (summer)  24.4 (autumn)  16.2 (winter) | Median 19 days | GPS tracking |
| Current study* | India | Continuous time movement models, AKDE (95% | 6.09 | Median 9 days | GPS tracking |

Table. S3. ΔAIC values for the activity range model comparisons for each individual dog.

| Dog ID | OU anisotropic | OUF anisotropic | OU | OUF | OUf anisotropic | IID anisotropic |
| --- | --- | --- | --- | --- | --- | --- |
| D1V1 | 0 | 2.009957 | 13.75997 | 15.76301 | 420.035957 | 2246.50817 |
| D2V2 | 0 | 2.006023 | 21.6967 | 23.6997 | 342.939535 | 1497.724371 |
| D3V2 | 0 | 2.001582 | 247.3654 | 249.3635 | 758.298947 | 3649.126524 |
| D4V2 | 0 | 1.998544 | 20.78438 | 22.77921 | 1211.907196 | 5141.972932 |
| D5V2 | 0 | 2.009705 | 32.2696 | 34.27499 | 58.811705 | 392.96917 |
| D6V1 | 0 | 2.004244 | 7.781406 | 9.776983 | 677.985195 | 2849.912699 |
| D7V2 | 0 | 2.022672 | 25.91456 | 111.8829 | 113.893971 | 203.612247 |
| D8V1 | 0 | 2.303314 | 96.33015 | 104.3857 | 177.875656 | 1122.994657 |
| D9V1 | 0 | 1.992732 | 21.40403 | 23.392 | 197.887928 | 351.337341 |
| D10V2 | 0 | 2.422494 | 11.09954 | 13.76486 | 977.961256 | 4103.394207 |
| D11V1 | 0 | 2.016833 | 25.11982 | 108.9501 | 110.959721 | 141.017389 |
| D12V1 | 0 | 2.004023 | 15.12266 | 17.12196 | 519.492014 | 1845.546518 |
| D13V1 | 0 | 1.965725 | 10.22088 | 12.17643 | 587.676573 | 1155.341411 |
| D14V1 | 0 | 2.010527 | 34.93571 | 36.9367 | 213.107512 | 646.151481 |
| D15V2 | 0 | 2.007764 | 128.2206 | 130.2247 | 133.282096 | 1017.879314 |


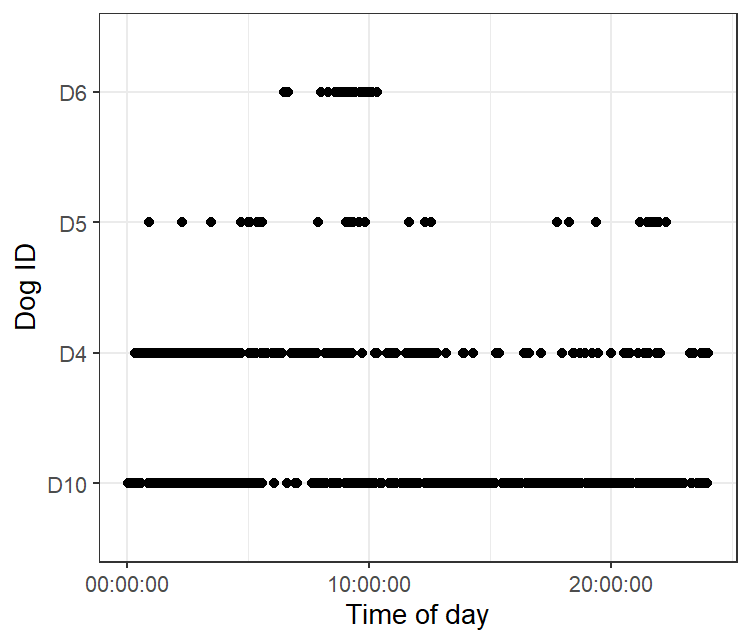


Figure S1. Time plots for GPS locations in the forest, illustrating individual temporal variation in dogs’ use of forest land. Here, D6 is a female sterilized shepherd’s dog, and D4 is a male unsterilized dog. D5 and D10 are female and male respectively, both sterilized. No other dogs utilised forest land in this study.


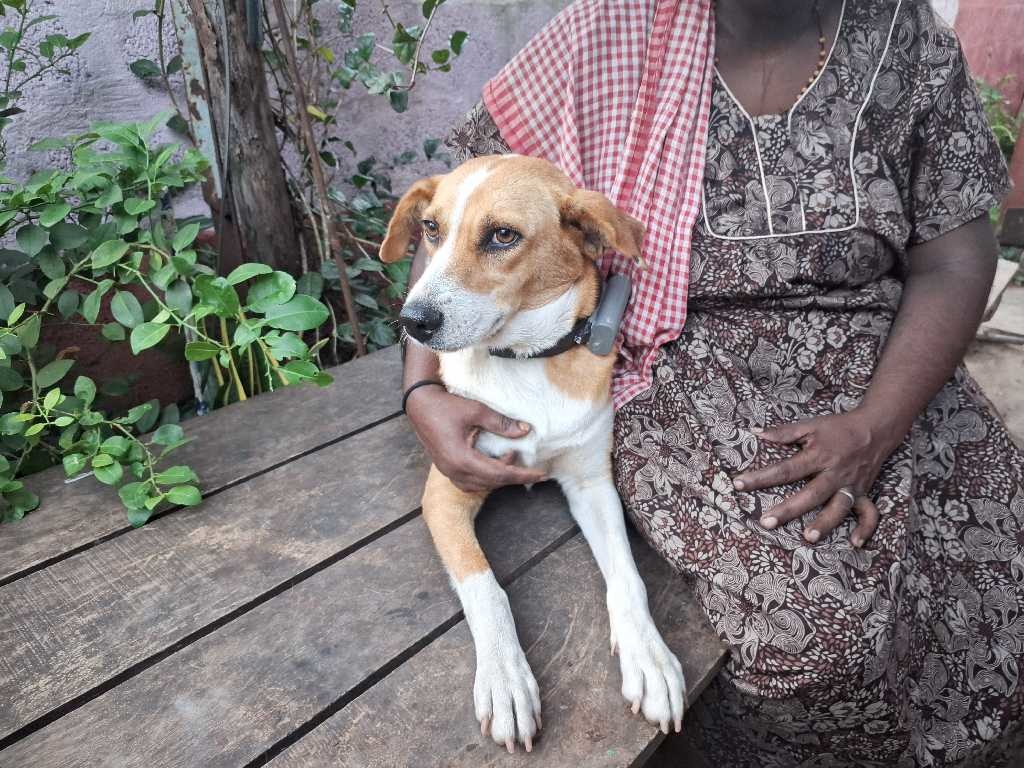


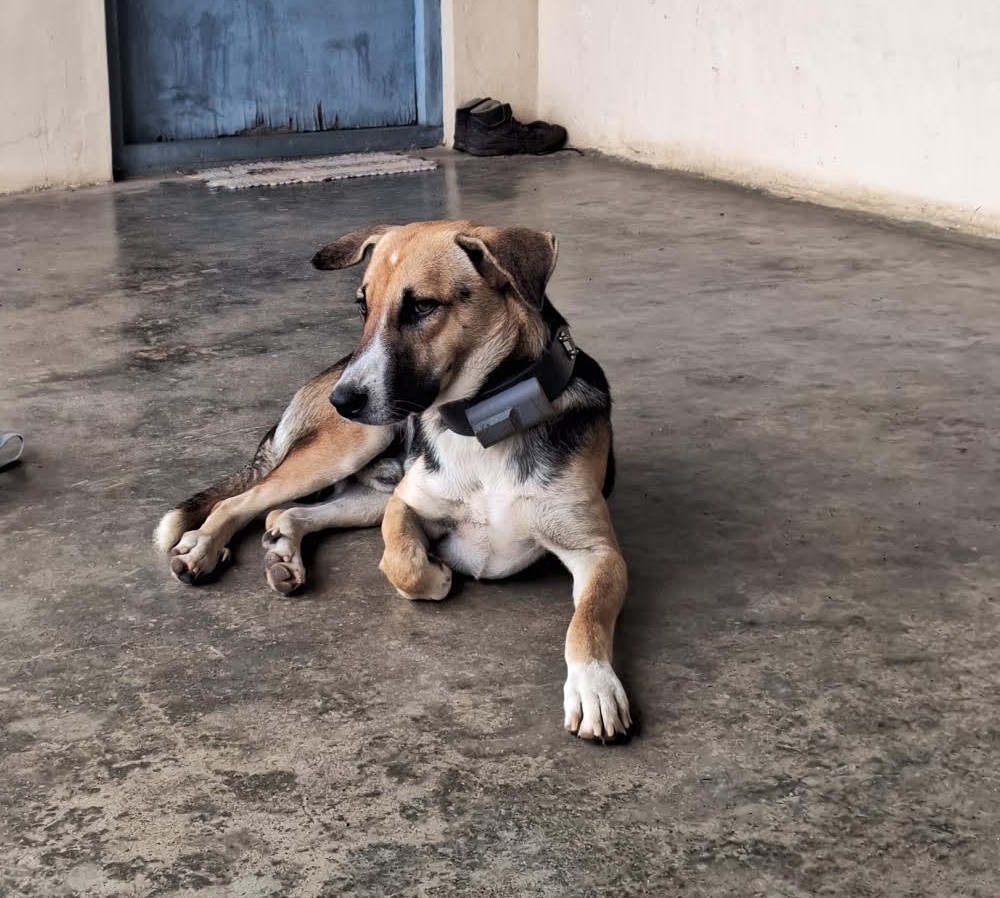


D


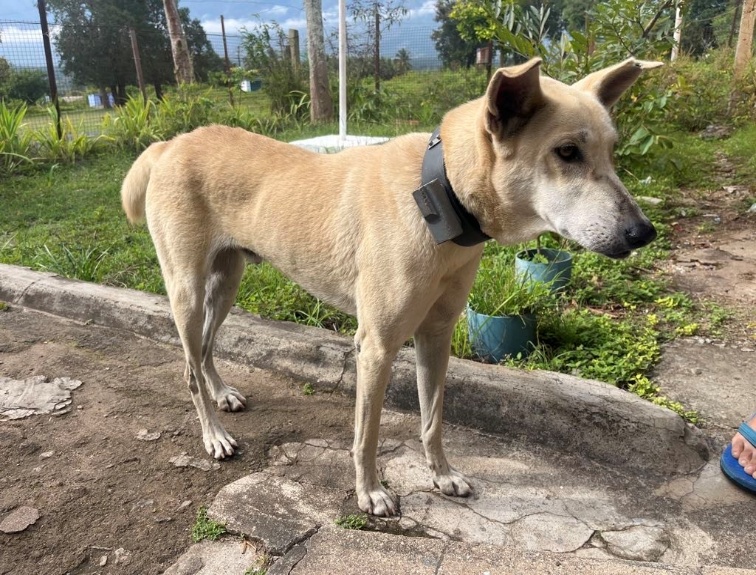


C


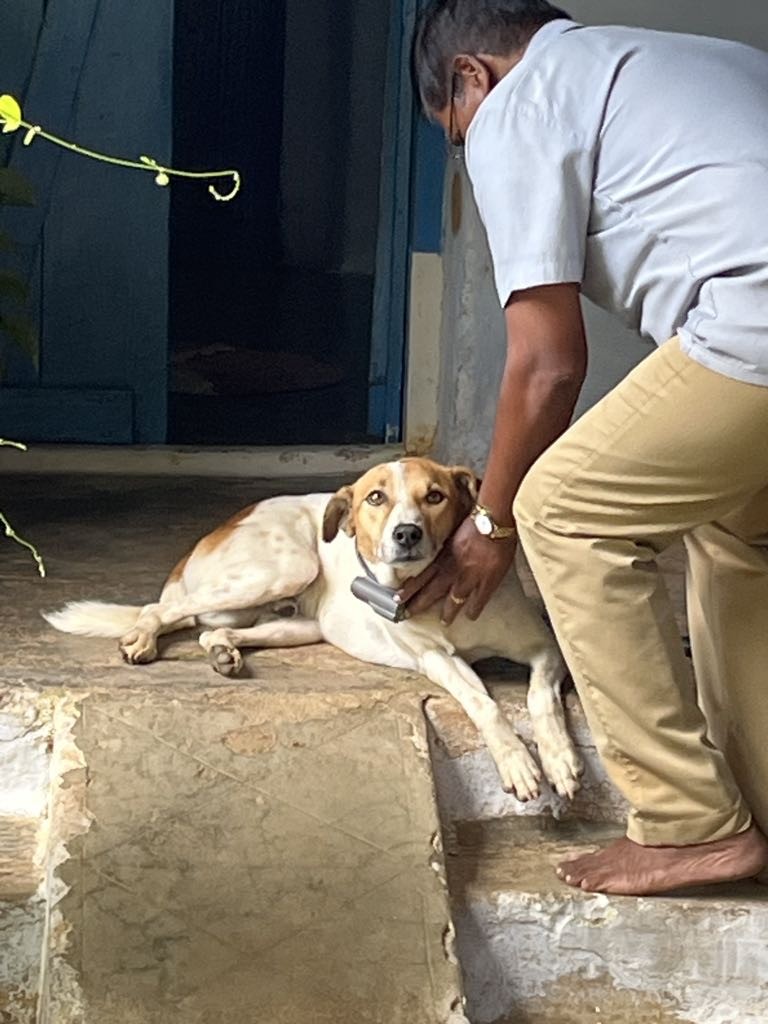


B


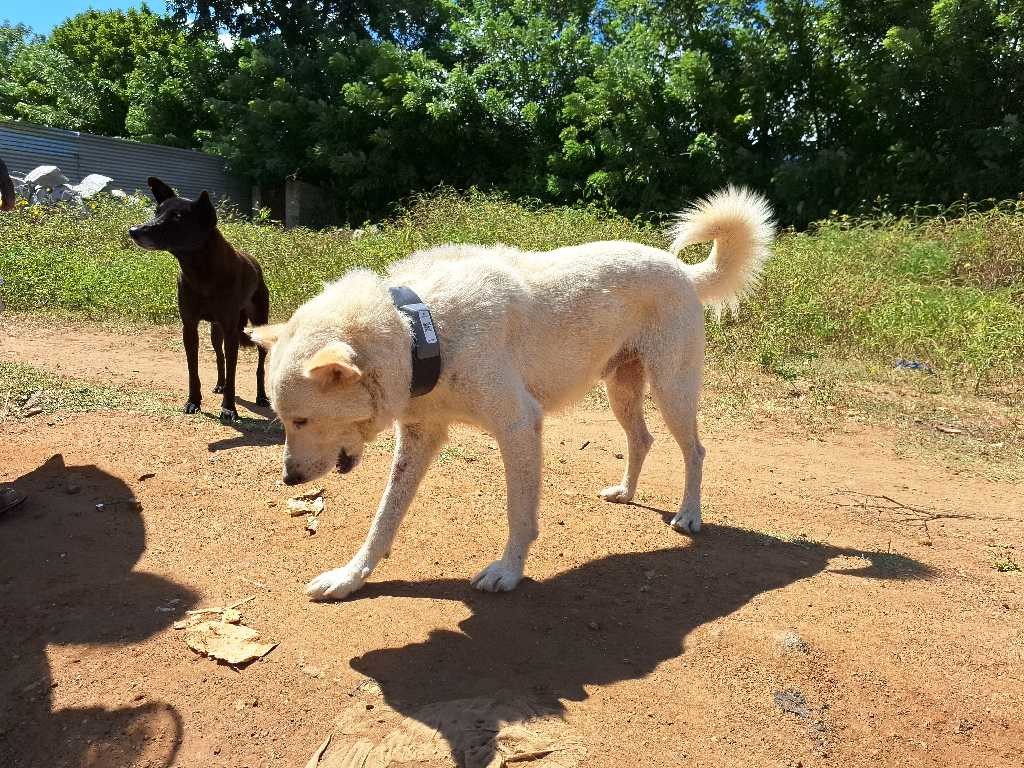


A


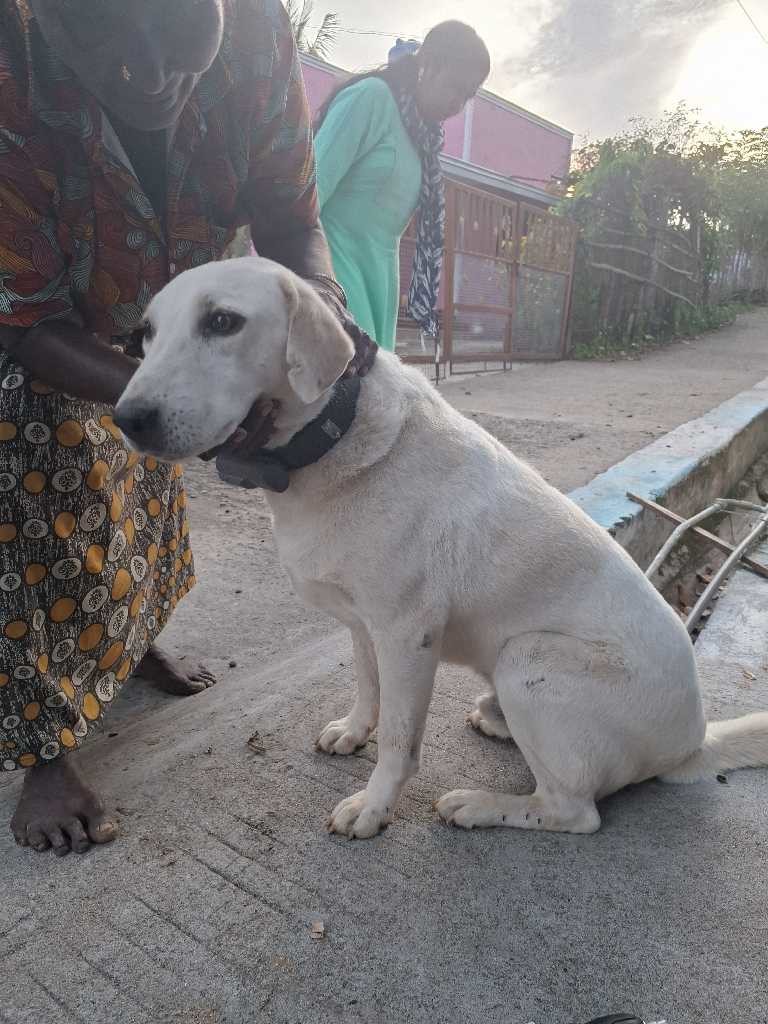


E


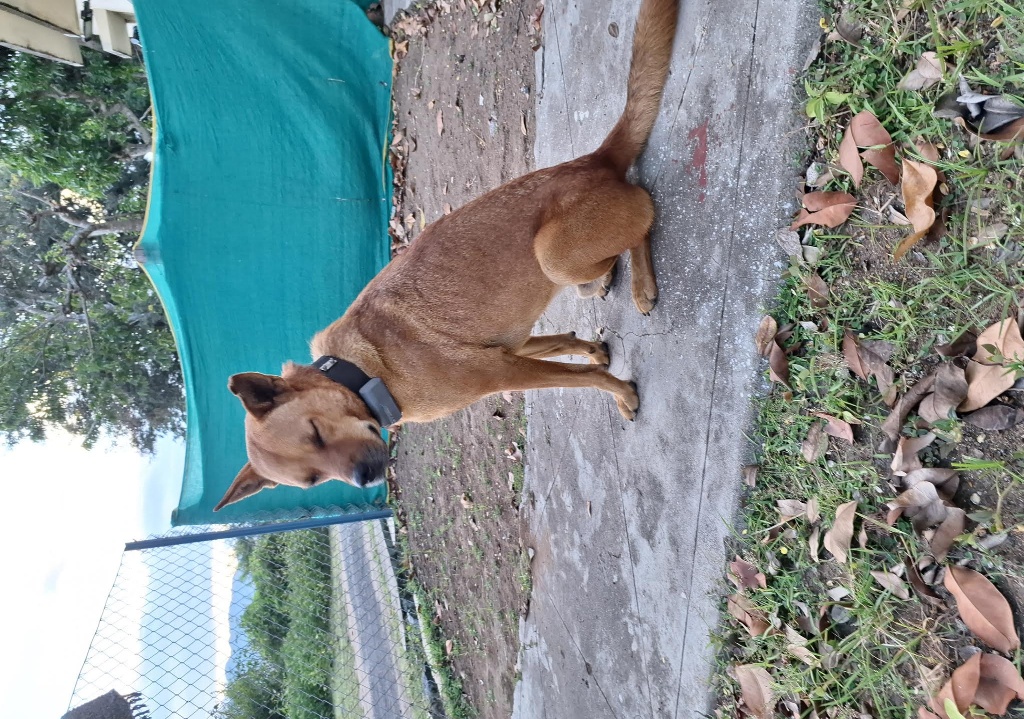


F

Fig S2. Photographs of collared dogs (A-F).

References (for table S2)

1. Meek PD. The movement, roaming behaviour and home range of free-roaming domestic dogs, Canis lupus familiaris, in coastal New South Wales. Wildl Res. 1999;26(6):847–55; doi:10.1071/wr97101.
2. Van Kesteren F, Mastin A, Mytynova B, Ziadinov I, Boufana B, Torgerson PR, et al. Dog ownership, dog behaviour and transmission of Echinococcus spp. in the Alay Valley, southern Kyrgyzstan. Parasitology. 2013 Nov;140(13):1674–84.
3. [Sparkes J, Körtner G, Ballard G, Fleming PJS, Brown WY. Effects of Sex and Reproductive State on Interactions between Free-Roaming Domestic Dogs. PLOS ONE. 2014 Dec 26;9(12):e116053; doi:10.1371/journal.pone.0116053.](https://www.zotero.org/google-docs/?KgUB47)
4. Dürr S, Ward MP. Roaming behaviour and home range estimation of domestic dogs in Aboriginal and Torres Strait Islander communities in northern Australia using four different methods. Preventive Veterinary Medicine. 2014 Nov 15;117(2):340–57.
5. [Ruiz-Izaguirre E, van Woersem A, Eilers K (c. ) H a. M, van Wieren SE, Bosch G, van der Zijpp AJ, et al. Roaming characteristics and feeding practices of village dogs scavenging sea-turtle nests. Anim Conserv. 2015;18(2):146–56; doi:10.1111/acv.12143.](https://www.zotero.org/google-docs/?KgUB47)
6. Kennedy B, Brown WY, Vernes K, Körtner G, Butler JRA. Dog and Cat Interactions in a Remote Aboriginal Community. Animals. 2018 May;8(5):65.
7. Muinde P, Bettridge JM, Sousa FM, Dürr S, Dohoo IR, Berezowski J, et al. Who let the dogs out? Exploring the spatial ecology of free-roaming domestic dogs in western Kenya. Ecol Evol. 2021;11(9):4218–31; doi:10.1002/ece3.7317.
8. Saavedra-Aracena L, Grimm-Seyfarth A, Schüttler E. Do dog-human bonds influence movements of free-ranging dogs in wilderness? Applied Animal Behaviour Science. 2021 Aug 1;241:105358.
9. Warembourg C, Wera E, Odoch T, Bulu PM, Berger-González M, Alvarez D, et al. Comparative Study of Free-Roaming Domestic Dog Management and Roaming Behavior Across Four Countries: Chad, Guatemala, Indonesia, and Uganda. Frontiers in Veterinary Science. 2021;8; doi: https://doi.org/10.3389/fvets.2021.617900
10. [Ladd R, Meek P, Eames JC, Leung LKP. Activity range and patterns of free-roaming village dogs in a rural Cambodian village. Wildl Res. 2023 Jul;51(1);](https://www.zotero.org/google-docs/?KgUB47) doi:10.1071/WR23024.
11. [Wilson-Aggarwal JK, Goodwin CED, Moundai T, Sidouin MK, Swan GJF, Léchenne M, et al. Spatial and temporal dynamics of space use by free-ranging domestic dogs Canis familiaris in rural Africa. Ecol Appl. 2021;31(5):e02328; doi:10.1002/eap.2328.](https://www.zotero.org/google-docs/?KgUB47)
12. Dürr S, Dhand NK, Bombara C, Molloy S, Ward MP. What influences the home range size of free-roaming domestic dogs? Epidemiology & Infection. 2017 May;145(7):1339–50.
13. Hudson EG, Brookes VJ, Dürr S, Ward MP. Domestic dog roaming patterns in remote northern Australian indigenous communities and implications for disease modelling. Prev Vet Med. 2017 Oct 1;146:52–60; doi:10.1016/j.prevetmed.2017.07.010.
14. [Molloy S, Burleigh A, Dürr S, Ward M. Roaming behaviour of dogs in four remote Aboriginal communities in the Northern Territory, Australia: preliminary investigations. Aust Vet J. 2017;95(3):55–63; doi:10.1111/avj.12562.](https://www.zotero.org/google-docs/?KgUB47)
15. Schüttler E, Saavedra-Aracena L, Jiménez JE. Spatial and temporal plasticity in free-ranging dogs in sub-Antarctic Chile. Applied Animal Behaviour Science. 2022;250:105610.
